# Up-Regulation of Imp3 Confers In Vivo Tumorigenicity on Murine Osteosarcoma Cells

**DOI:** 10.1371/journal.pone.0050621

**Published:** 2012-11-30

**Authors:** Arisa Ueki, Takatsune Shimizu, Kenta Masuda, Sayaka I. Yamaguchi, Tomoki Ishikawa, Eiji Sugihara, Nobuyuki Onishi, Shinji Kuninaka, Keita Miyoshi, Akihiro Muto, Yoshiaki Toyama, Kouji Banno, Daisuke Aoki, Hideyuki Saya

**Affiliations:** 1 Division of Gene Regulation, Institute for Advanced Medical Research, School of Medicine, Keio University, Tokyo, Japan; 2 Department of Obstetrics and Gynecology, School of Medicine, Keio University, Tokyo, Japan; 3 Department of Pathophysiology, School of Pharmacy and Pharmaceutical Sciences, Hoshi University, Tokyo, Japan; 4 Department of Orthopedic Surgery, School of Medicine, Keio University, Tokyo, Japan; 5 Kasai R&D Center, Daiichi Sankyo Co. Ltd., Tokyo, Japan; 6 Department of Molecular Biology, School of Medicine, Keio University, Tokyo, Japan; 7 Japan Science and Technology Agency, Core Research for Evolutional Science and Technology (CREST), Tokyo, Japan; National Cancer Center Research Institute, Japan

## Abstract

Osteosarcoma is a high-grade malignant bone tumor that manifests ingravescent clinical behavior. The intrinsic events that confer malignant properties on osteosarcoma cells have remained unclear, however. We previously established two lines of mouse osteosarcoma cells: AX cells, which are able to form tumors in syngeneic mice, and AXT cells, which were derived from such tumors and acquired an increased tumorigenic capacity during tumor development. We have now identified Igf2 mRNA-binding protein3 (Imp3) as a key molecule responsible for this increased tumorigenicity of AXT cells in vivo. Imp3 is consistently up-regulated in tumors formed by AX cells, and its expression in these cells was found to confer malignant properties such as anchorage-independent growth, loss of contact inhibition, and escape from anoikis in vitro. The expression level of Imp3 also appeared directly related to tumorigenic ability in vivo which is the critical determination for tumor-initiating cells. The effect of Imp3 on tumorigenicity of osteosarcoma cells did not appear to be mediated through Igf2-dependent mechanism. Our results implicate Imp3 as a key regulator of stem-like tumorigenic characteristics in osteosarcoma cells and as a potential therapeutic target for this malignancy.

## Introduction

Malignant tumors are derived from transformed normal cells. As the disease course progresses, tumor cells acquire various malignant biological properties such as deregulated cell proliferation, anchorage-independent growth, increased invasiveness, as well as the potential to induce neovascularization and to undergo metastasis, the combination of all of which eventually becomes life threatening [1], [2]. The cell-intrinsic molecular events that underlie the conversion of tumor cells from initial relatively benign state to high-grade malignant state remain largely unknown, however, as does whether master regulators of such malignant properties exist.

We previously established a line of mouse osteosarcoma cells, designated AX, through overexpression of c-MYC in bone marrow stromal cells derived from Ink4a and Arf knockout mice. Subcutaneous injection of AX cells into syngeneic mice resulted in the formation of lethal osteosarcoma tumors that underwent metastasis, mimicking the pathology of human osteosarcoma [3]. We further established tumor-initiating cells, designated AXT, from such AX cell-derived subcutaneous tumors. Injection of AXT cells resulted in the generation of tumors that were identical histologically to those formed by AX cells but with a greatly shortened disease course, suggesting that tumorigenic capability of AXT cells increased during initial tumor formation in vivo. Further investigation revealed that AXT cells showed enhanced anchorage-independent growth and anoikis resistance compared with AX cells.

Anchorage-independent growth and anoikis resistance, which reflect the ability of cells undergoing continuous proliferation and avoiding death after loss of contact with the extracellular matrix, have been found to correlate with transformation, tumorigenic activity, tumor progression, and metastasis [1], [4]. Molecules that confer these properties on cancer cells have remained to be definitively identified, however. We have now compared the gene expression profiles of AX and AXT cells and have identified the gene for Imp3 as being highly overexpressed in AXT cells. We further found that Imp3 plays a key role in the anchorage-independent growth and anoikis resistance in vitro as well as in their tumorigenicity in vivo. Our findings thus indicate that Imp3 is a potential target for therapeutic control of the aggressiveness of osteosarcoma.

## Materials and Methods

### Cell Culture

Mouse osteosarcoma AX and AXT cells were established as previously described [3] and were cultured in DMEM High Glucose (Invitrogen, Carlsbad, CA) supplemented with 10% FBS and antibiotic-antimycotic (100 U/ml, Invitrogen). In the experiments of inhibition of DNA methyltransferase and/or histone deacetylase, AX cells were treated with 5-AZA-2′-DEOXYCYTIDINE (5AzaD) (Sigma-Aldrich, St. Louis, MO), TRICHOSTATIN A (TSA) (SIGMA), Valproic acid (VPA) (SIGMA) or SAHA (SIGMA) at the indicated concentration for one day. Cells were collected and subjected to RT and real-time PCR analysis.

### RT and Real-time PCR Analysis

Total RNA was extracted from cells or tumors with the use of RNeasy Mini Spin columns (Qiagen, Hilden, Germany) and was subjected to RT with a Prime Script RT-PCR kit (Takara, Shiga, Japan). Real-time PCR analysis was performed with SYBR Premix Ex TaqII and Thermal Cycler Dice (Takara). The sequences of primers are shown in Table S1. Data were normalized by the corresponding amount of *Gapdh* mRNA and are means ± SD for three independent experiments.

### Immunostaining

Immunohistochemical analysis was performed according to standard methods. Deparaffinized sections were stained with rabbit polyclonal antibodies to GFP-FL (Santa Cruz Biotechnology, Santa Cruz, CA) or IMP3 (MBL, Aichi, Japan). Immune complexes were detected with Histofine (Nichirei Bioscience, Tokyo, Japan) and Simple Stain kit (Nichirei Bioscience). For immunofluorescence analysis, cells were fixed with acetone and stained with primary antibodies and Alexa546-conjugated secondary antibodies (Invitrogen). Nuclei were stained with TOTO3 (Invitrogen). Samples were observed with LSM510 confocal microscope (Zeiss, Gottingen, Germany) and analyzed with LSM image browser (Zeiss).

### Human Osteosarcoma Tissue Array

An array of human osteosarcoma specimens was obtained from Folio Biosciences (Powell, OH) and was subjected to immunohistochemical staining for IMP3 as described above.

### Gene Expression Profiling

Gene expression profiling was performed with a 3D-DNA chip (Toray, Tokyo, Japan) as previously described [3].

### Knockdown of Imp3 and Igf2

AXT cells were infected with the pRePS retroviral vector (kindly provided by T. Hara) as previously described [5] and were then subjected to selection in the presence of puromycin (3 µg/ml). The sequences of the sense oligonucleotides for Imp3 and Igf2 shRNAs are shown in Table S1.

### Plasmid and Retroviral Gene Transfer

Mouse Imp3 cDNA was isolated from a cDNA library of AXT cells and cloned into the PMXs-IP retroviral plasmid (kindly provided by T. Kitamura). Retroviral gene transfer was performed as previously described [6]. Infected AX cells were subjected to selection in the presence of puromycin (3 µg/ml).

### Flow Cytometry

Cells were stained with FITC-conjugated annexin V and propidium iodide (PI) with use of apoptosis detection kit (BD Biosciences, Franklin Lakes, NJ) and were analyzed (10,000 cells per sample) with FACS Calibur (BD Biosciences).

### Cell Proliferation Assay

Cells (1000 per well) were transferred to 96-well tissue culture plates (BD Biosciences) or 96-well ultra low-adherence plates (Corning, NY, USA) and were cultured in DMEM supplemented with 10% FBS and in the absence or presence of mouse Igf2 (R&D Systems, Mineapolis, MN) as indicated. Cell proliferation was assayed in triplicate with the use of a Cell Titer Glo assay kit (Promega, Madison, WI). Quantitative data are expressed relative to the value for time 0 and are means ± SD for three independent experiments.

### Immunoblot Analysis

Cells were lysed with Laemmli sample buffer (BioRad, Hercules, CA) and subjected to immunoblot analysis according to standard procedures. Primary antibodies included those to IMP3 (MBL), α-Tubulin (SIGMA), rpS6 (Cell Signaling Technology, Beverly, MA), and Ago2 (Wako, Osaka, Japan).

### Tumor Xenograft Model

Single-cell suspensions were prepared in 100 µl of PBS and were injected subcutaneously (1×10^6^ cells; bilaterally) or intraperitoneally (3×10^6^ cells) into 8-week-old syngeneic female C57BL/6 mice. The weight of subcutaneous tumors was measured after 28 days unless indicated otherwise.

### Polysome Analysis

AXT cells were cultured in 10-cm dishes, washed with ice-cold PBS, and lysed in a solution containing 20 mM Hepes–KOH (pH7.4), 150 mM NaCl, 2.5 mM MgCl_2_, 0.1% NP-40, 1 mM DTT and protease inhibitors (Roche, Mannheim, Germany). The lysate was subjected to centrifugation at 15,000×*g* for 10 min at 4°C, and the resulting supernatant was applied to 5–30% (w/v) sucrose density gradient prepared in the cell lysis buffer. The gradient was then centrifuged at 40,000 r.p.m for 90 min at 4°C, after which the absorbance profile at 254 nm was recorded with PGF fractionator (BIOCOMP, Fredericton, NB, Canada) and gradient fractions were subjected to immunoblot analysis.

### Statistical Analysis

Quantitative data are presented as means ± SD and were compared with Student’s *t* test. Kaplan-Meier survival curves were compared with the log-rank test. The relation between tumor weight and *Igf2* expression was evaluated by calculation of the correlation coefficient (CC).

### Ethics Statement

Animal care and procedures were performed in accordance with the guidelines of Keio University. The ethics committee of Keio University specifically approved this study.

## Results

### Up-regulation of Imp3 Expression in AX Cells During Tumor Formation in vivo

**Figure 1:**
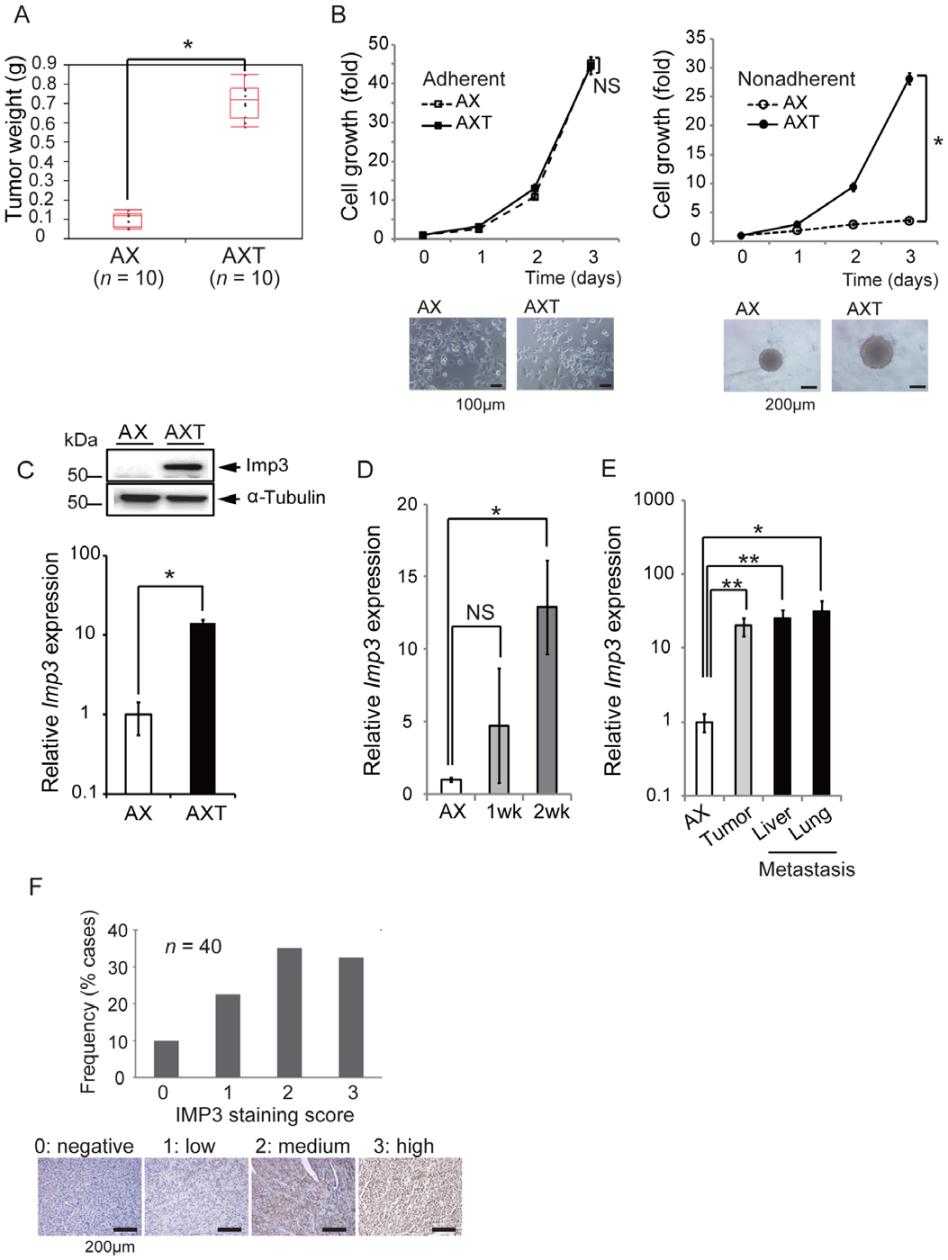
Up-regulation of Imp3 expression during tumor formation in vivo. (**A**) AX or AXT cells were injected bilaterally and subcutaneously into syngeneic mice, and the weight of the tumors was measured. *P<0.0001. (**B**) Cell proliferation assays for AX or AXT cells cultured under adherent or nonadherent conditions. *P<0.001. NS, not significant. Representative bright-field images of cells after culture for 2 days are shown. (**C**) Immunoblot analysis of Imp3 expression in AX and AXT cells. α-Tubulin was examined as a loading control. Real-time PCR analysis of *Imp3* expression in AX and AXT cells. *P<0.01. (**D**, **E**) Real-time PCR analysis of *Imp3* expression in subcutaneous AX cells at 1 or 2 weeks after injection into mice, and in primary tumors and metastatic lesions formed by AX cells. *P<0.05, **P<0.01. NS, not significant. (**F**) Immunohistochemical staining of IMP3 in human osteosarcoma samples. The intensity of staining was scored from 0 to 3. Representative images are shown.

Consistent with our previous observations [3], the weight of tumors formed after subcutaneous cell injection in C57BL/6 syngeneic mice was significantly greater for AXT cells than for AX cells (Figure 1A). Whereas AX and AXT cells exhibited similar growth rates when cultured in normal tissue culture plates, the growth rate of AXT cells was markedly greater than that of AX cells under non-adherent culture conditions (Figure 1B). These findings suggested that AXT cells acquired properties of anchorage-independent growth and anoikis resistance, and that these properties might contribute to their increased tumorigenicity in vivo.

To explore the molecular events underlying the conversion of AX cells into AXT cells, we compared the gene expression profiles of these cells. The gene expression patterns were largely similar (data not shown), but we extracted genes for which the AXT/AX log_2_ ratio of normalized expression values was ≥3.0 (Table S2). From among the 38 identified genes, we selected candidate molecules for further evaluation according to the following criteria: The expression level (1) is low in normal tissue; (2) is associated with poor prognosis in various human malignancies; (3) increases during tumor development from AX cells; and (4) is directly related to tumorigenic activity in vivo as revealed by forced expression of the encoded protein in AX cells and its depletion in AXT cells. We found that Imp3 meets all these criteria, as shown below.

Imp3 is expressed predominantly during embryogenesis and in various tumors [7]–[14], with its expression being limited to the placenta and testis in normal adult mice [9]. Imp3 is thus considered an oncofetal protein and is highly expressed in various human malignancies [8], [9], [15], [16]. The abundance of Imp3 was markedly higher in AXT cells than in AX cells and *Imp3* expression in AXT cells was >10 times that in AX cells (Figure 1C). Of note, the amount of *Imp3* mRNA in AX cells after inoculation into syngeneic mice increased in a time-dependent manner (Figure 1D), and it was significantly higher in both primary and metastatic lesions than in parental AX cells (Figure 1E). These results thus suggested that *Imp3* expression in AX cells is maintained at low level in vitro but is up-regulated during tumor formation in vivo in association with the conversion of AX cells into highly tumorigenic AXT cells.

### IMP3 Expression in Human Osteosarcoma

Given that Imp3 expression appeared to be associated with an aggressive phenotype of mouse osteosarcoma, we examined the expression in human osteosarcoma. Immunohistochemical analysis of a tissue array containing 40 human osteosarcoma samples showed that IMP3 was expressed in 36 (90%) of the specimens (Figure 1F). Scoring of staining intensity from 0 to 3 revealed a high expression level (score of 2 or 3) in 27 of the 40 samples (67.5%), suggesting that deregulation of IMP3 expression occurs frequently in human osteosarcoma.

### Up-regulation of Imp3 Expression at the Clonal Level in vivo and its Relation to Tumorigenic Activity

**Figure 2:**
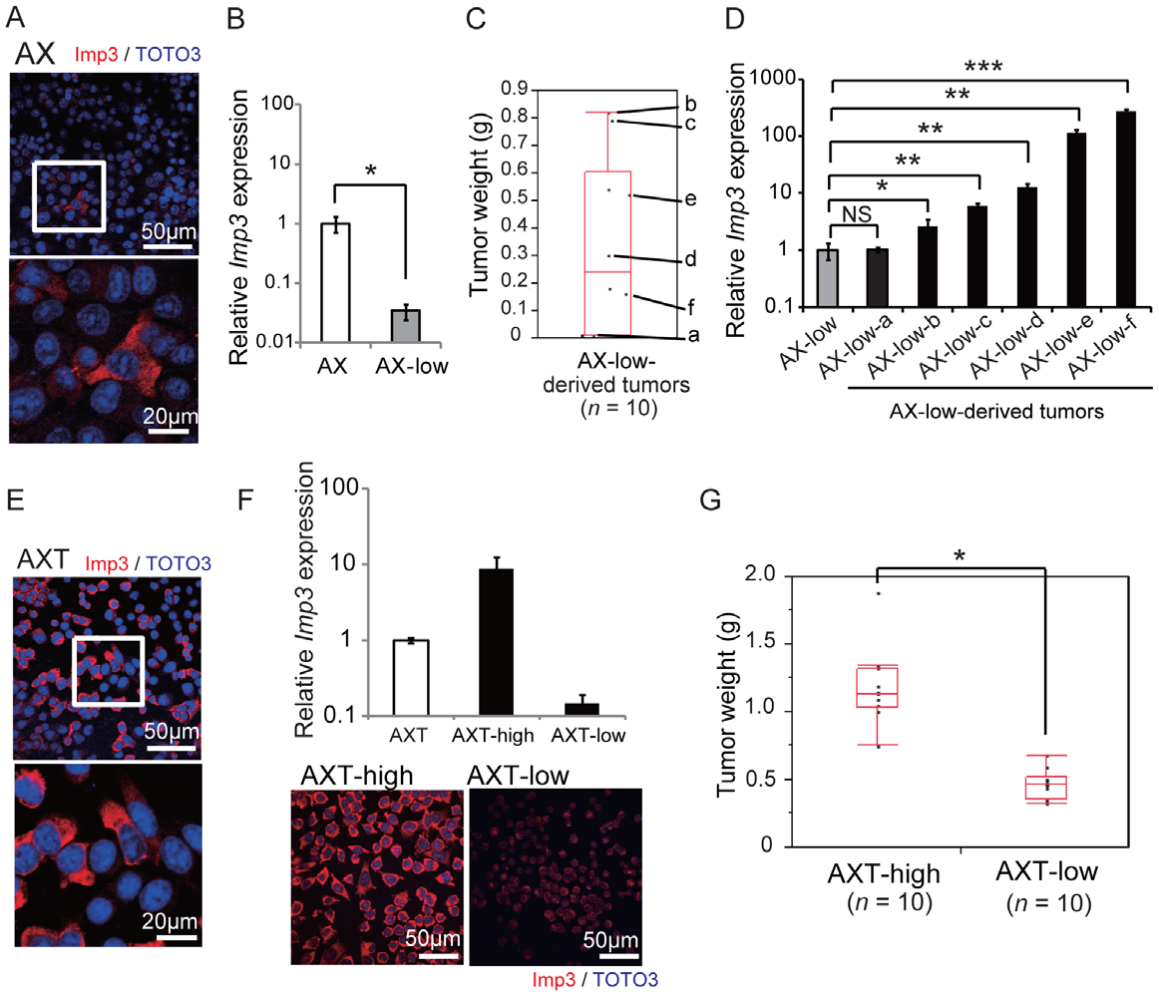
Tumorigenic activity of osteosarcoma cells correlates with Imp3. (**A**) Immunofluorescence analysis of Imp3 expression in AX cells. The boxed region is shown at higher magnification in the lower panel. (**B**) Real-time PCR analysis of *Imp3* expression in AX cells and in an AX subclone (designated AX-low) obtained by single-cell cloning. *P<0.01. (**C**) Weight of tumors derived from subcutaneously injected AX-low cells. (**D**) Real-time PCR analysis of *Imp3* expression in AX-low cells as well as in the AX-low cell-derived tumors. *P<0.05, **P<0.01, ***P<0.001. NS, not significant. (**E**) Immunofluorescence analysis of Imp3 expression in AXT cells. The boxed region is shown at higher magnification in the lower panel. (**F**) AXT subclones (designated AXT-high and AXT-low) isolated by single-cell cloning were subjected to real-time PCR analysis of *Imp3* as well as to immunofluorescence analysis of Imp3 protein. (**G**) Weight of tumors derived from subcutaneously injected AXT-high or AXT-low cells. *P<0.001.

Given that a small fraction of AX cells (2.6±0.27%) was found to express Imp3 at a relatively high level in culture (Figure 2A), we examined whether these few cells might preferentially expand and generate tumors in vivo or whether Imp3 expression becomes up-regulated during tumor formation. We performed single-cell cloning of AX cells and isolated the clone with the lowest *Imp3* expression (designated AX-low), which was only ∼3% of that in the original AX cells (Figure 2B). Subcutaneous injection of AX-low cells resulted in the formation of tumors of various sizes (Figure 2C). We then examined the expression of *Imp3* in these tumor cells by establishing sublines after mechanical dissection and mincing of tumor tissues. Although the *Imp3* expression in AX-low-a cells, which were established from the smallest tumor, was virtually identical to that in the parental AX-low cells, other established cells from larger tumors showed significantly higher level of *Imp3* expression (Figure 2D). These results indicated that AX clones that originally exhibit low level of *Imp3* expression in vitro can become cells that express *Imp3* at high level during tumor formations in vivo.

We tried to gain insight into the molecular mechanisms related to the up-regulation of *Imp3* in AX cells during tumorigenesis in vivo. To examine whether the expression of *Imp3* could be epigenetically regulated, AX cells were treated with DNA methyltransferase inhibitor; 5AzaD and histone deacetylase inhibitors; TSA, VPA and SAHA. Treatment of these epigenetic modification agents in AX cells for one day resulted in significant up-regulation of *Imp3* expression, albeit the effect of 5AzaD, VPA or SAHA was modest. Moreover, the combination of 5AzaD and TSA showed additive effect (Figure S1). These findings suggest that the up-regulation of *Imp3* during tumorigenesis in AX cells is at least partially attributable to the epigenetic regulation such as DNA methylation and histone acetylation.

AXT cells were also heterogeneous in terms of the expression level of Imp3 (Figure 2E). We therefore performed single-cell cloning of AXT cells to examine the relation between *Imp3* expression and tumorigenic potential. We isolated clones showing the highest and lowest levels of *Imp3* expression (designated AXT-high and AXT-low, respectively), with the abundance of *Imp3* mRNA in the former being more than nine times and that in the latter being one-sixth of that in parental AXT cells (Figure 2F). The tumors formed after subcutaneous injection of AXT-high cells were larger than those formed by AXT-low cells (Figure 2G), suggesting that *Imp3* expression is directly related to the tumorigenic activity of osteosarcoma cells.

### Overexpression of Imp3 in AX Cells Confers High Tumorigenic Activity

**Figure 3:**
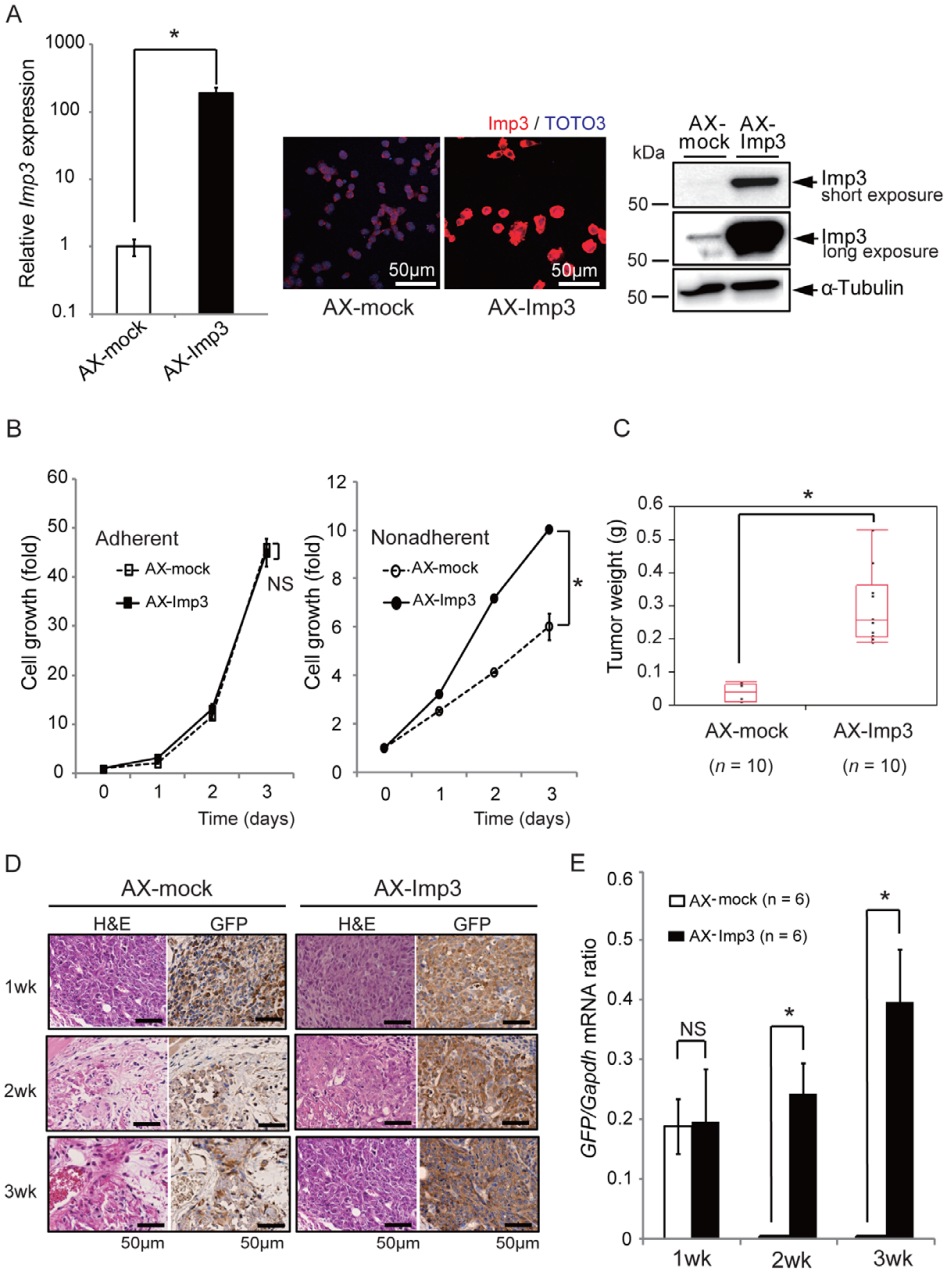
Imp3 overexpression in AX cells promotes cell proliferation and tumorigenic activity. (**A**) The expression level of Imp3 was evaluated by real-time PCR analyses, immunofluorescence and immunoblotting. *P<0.01. (**B**) Cell proliferation assays for AX-mock and AX-Imp3 cells cultured under adherent or nonadherent conditions. *P<0.01. NS, not significant. (**C**) Weight of tumors derived from subcutaneously injected AX-mock or AX-Imp3 cells. *P<0.001. (**D, E**) Tumors formed at 1, 2, or 3 weeks after subcutaneous injection of AX-mock or AX-Imp3 cells in mice were subjected to H&E staining and to immunohistochemical staining for GFP in serial sections as well as to real-time PCR analysis of *GFP.* *P<0.01. NS, not significant.

We next evaluated whether forced expression of Imp3 might affect the tumorigenic activity of AX cells. We generated AX cells that stably overexpress Imp3 (designated AX-Imp3 cells) by retroviral gene transfer (Figure 3A). Whereas AX-Imp3 and control infected cells (designated AX-mock cells) showed similar growth patterns under normal culture conditions, the proliferation rate of AX-Imp3 cells was markedly greater than that of the control cells under non-adherent conditions (Figure 3B), similar to the difference observed between AXT and AX cells (Figure 1B). Examination of tumorigenicity in vivo revealed that the tumors formed by AX-Imp3 cells being significantly larger than those formed by AX-mock cells (Figure 3C). We estimated the proportion of live tumor cells by immunohistochemical staining for GFP (Figure 3D) as well as by real-time PCR analysis of *GFP* mRNA (Figure 3E), given that AX cells were engineered to express GFP. One week after cell injection, both cells showed similar patterns of GFP expression and amounts of *GFP* mRNA, suggesting similar proportions of live cells. However, at 2 or 3 weeks after cell injection, the proportion of GFP-positive cells and the amount of *GFP* mRNA had declined for AX-mock but not for AX-Imp3 (Figure 3D, E). Collectively, these results indicated that overexpression of Imp3 conferred growth advantage on osteosarcoma cells under stressful conditions represented by loss of matrix attachment and thereby increased their tumorigenic activity in vivo.

### Knockdown of Imp3 Attenuates the Malignant Phenotype of AXT Cells in vitro

**Figure 4:**
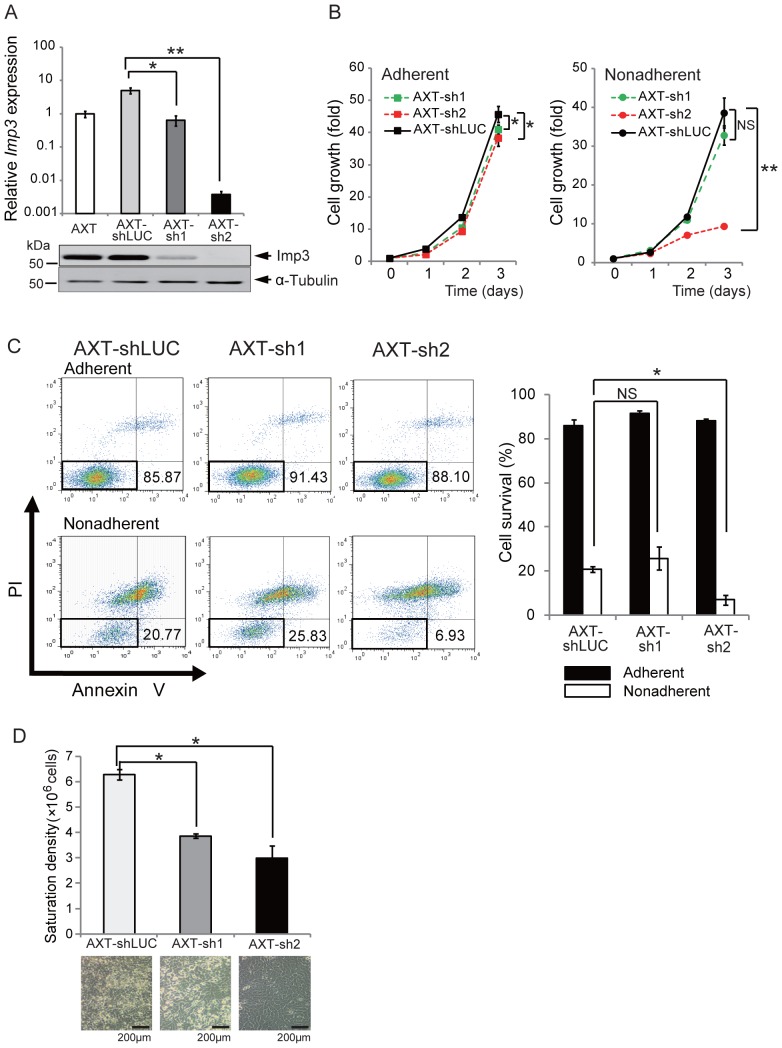
Knockdown of Imp3 in AXT cells attenuates malignant cellular phenotype. (**A**) The efficacy of knockdown of Imp3 in AXT cells was evaluated by real-time PCR analyses and immunoblotting. *P<0.01, **P<0.001. (**B**) Cell proliferation assays for AXT-shLUC, AXT-sh1, and AXT-sh2 cells cultured under adherent or nonadherent conditions. *P<0.05, **P<0.001. NS, not significant. (**C**) AXT-shLUC, AXT-sh1, and AXT-sh2 cells were cultured under adherent (upper panels) or nonadherent (lower panels) conditions for 24 h and were then stained with annexin V and PI. The percentages of viable (annexin V– and PI-negative) cells are indicated for a representative experiment. The percentage of viable cells was determined as means ± SD for three independent experiments. *P<0.01. NS, not significant. (**D**) AXT-shLUC, AXT-sh1, and AXT-sh2 cells were cultured under normal conditions for 3 days, after which the saturation density was determined by counting total cell number. *P<0.01. Representative bright-field images of cells after culture for 3 days are also shown.

To evaluate the relation between Imp3 expression and tumorigenic activity, we depleted AXT cells of Imp3 by shRNAs targeting two different coding sequences (yielding AXT-sh1 and AXT-sh2 cells). The amount of *Imp3* mRNA was reduced by a factor of ∼1000 or ∼7 in AXT-sh2 and AXT-sh1 cells, respectively, compared with cells expressing control shRNA (AXT-shLUC cells), with similar changes also being apparent for Imp3 protein (Figure 4A). Whereas knockdown of Imp3 resulted in only a small reduction in the rate of AXT cell proliferation under normal culture conditions, the growth rate of AXT-sh2 cells was greatly reduced compared with that of AXT-shLUC cells under non-adherent conditions (Figure 4B). AXT-sh2 cells thus showed growth characteristics similar to those of AX cells (Figure 1B). In contrast, the growth rate of AXT-sh1 cells did not differ significantly from the control cells under non-adherent conditions (Figure 4B), likely as a result of the limited knockdown of Imp3 in AXT-sh1 cells.

We next investigated anchorage-independent survival of AXT-shLUC and Imp3-depleted AXT cells. Flow cytometric analysis of cells stained with annexin V and propidium iodide (PI) revealed that the size of the double-negative (viable) population was equally large for each cell line under adherent conditions (Figure 4C). However, under non-adherent conditions, the size of the viable population was significantly smaller for AXT-sh2 than for AXT-shLUC cells. Again, similar to the cell growth pattern, the anchorage-independent survival of AXT-sh1 cells did not differ significantly from the control cells. These findings suggested that the up-regulation of Imp3 expression in tumor cells might contribute to their escape from anoikis.

Loss of contact inhibition and consequent overgrowth to a high density is key malignant properties of transformed cells [1], [17]. We cultured AXT-shLUC and Imp3-depleted AXT cells to confluence and determined the saturation density. Whereas AXT-shLUC cells continued to grow past confluence, resulting in the formation of large piles and high saturation density, AXT-sh1 and AXT-sh2 cells manifested contact inhibition and lower saturation density (Figure 4D). Aberrant Imp3 expression in tumor cells may thus promote anchorage-independent growth and loss of contact inhibition.

### Knockdown of Imp3 in AXT Cells Suppresses Tumorigenic Activity in vivo

**Figure 5:**
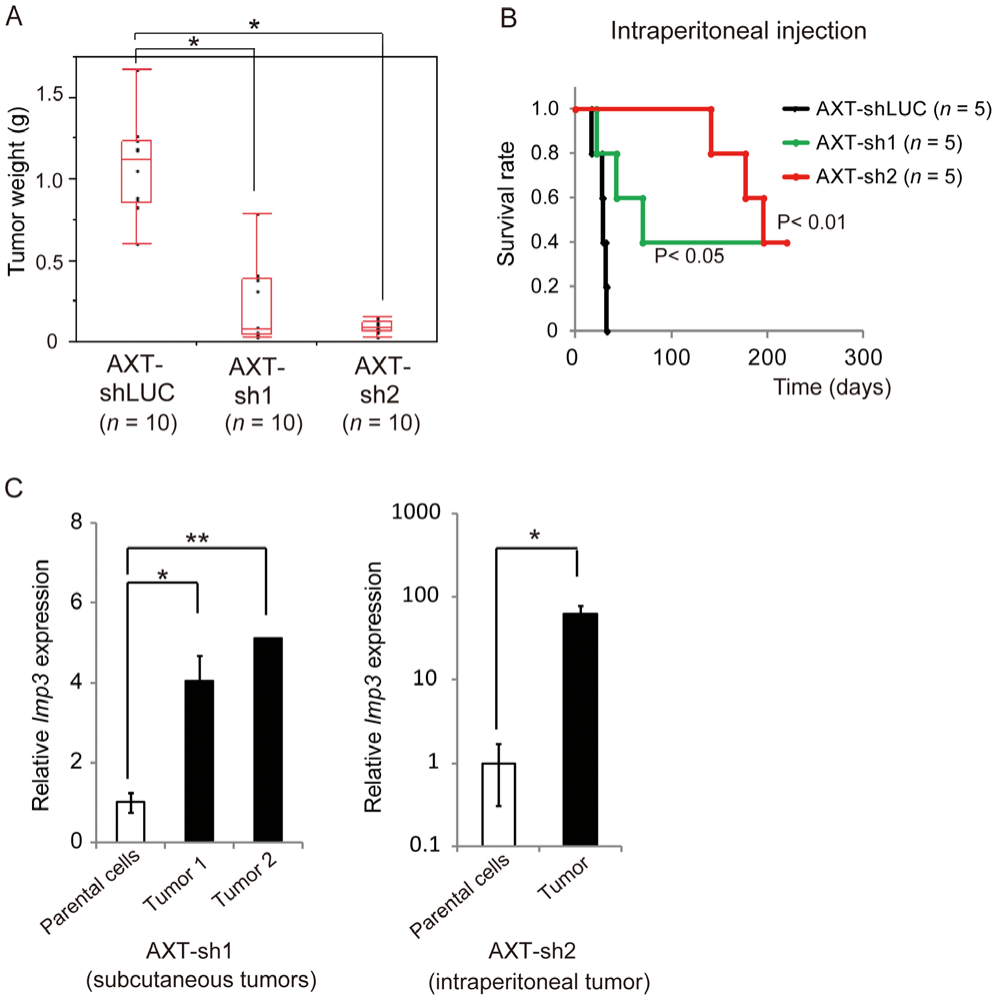
Knockdown of Imp3 in AXT cells suppresses tumorigenic activity in vivo. (**A**) Weight of tumors formed in syngeneic mice after subcutaneous injection of AXT-shLUC, AXT-sh1, or AXT-sh2 cells. *P<0.001. (**B**) Kaplan-Meier survival analysis of mice injected intraperitoneally with AXT-shLUC, AXT-sh1, or AXT-sh2 cells. P values for comparison with AXT-shLUC were determined by the log-rank test. (**C**) Real-time PCR analysis of *Imp3* expression in lethal osteosarcoma tumors at 49 days after bilateral subcutaneous injection of AXT-sh1 cells (left panel) or at 141 days after intraperitoneal injection of AXT-sh2 cells (right panel). *P<0.01, **P<0.001.

We next examined whether knockdown of Imp3 in AXT cells might affect tumorigenic activity in vivo. Whereas all mice injected subcutaneously with Imp3-depleted AXT cells developed palpable tumors, the weight of these tumors was significantly smaller than those derived from AXT-shLUC cells (Figure 5A). None of the mice injected with Imp3-depleted AXT cells manifested lung or liver metastasis, whereas all mice injected with AXT-shLUC cells developed metastases at both sites (Table S3). We also evaluated tumorigenic activity after intraperitoneal injection of osteosarcoma cells, which resulted in earlier death from primary tumors. Whereas AXT-sh2 cells did not generate lethal tumors within >140 days, AXT-shLUC cells did so within 33 days (Figure 5B). One mouse injected subcutaneously with AXT-sh1 cells developed lethal osteosarcoma tumors at 49 days after cell injection, and the *Imp3* expression in these tumors was markedly increased compared with the parental AXT-sh1 cells (Figure 5C: left panel). Furthermore, *Imp3* expression in a lethal tumor derived from AXT-sh2 cells at 141 days after intraperitoneal cell injection was also increased compared with the parental cells (Figure 5C: right panel). The efficiency of Imp3 knockdown by shRNA might be reduced during tumor development with a long latency. Together, these findings indicated that up-regulation of Imp3 in osteosarcoma cells plays an important role in tumorigenesis in vivo.

### Imp3 Regulates Tumorigenic Activity Independently of Igf2

**Figure 6:**
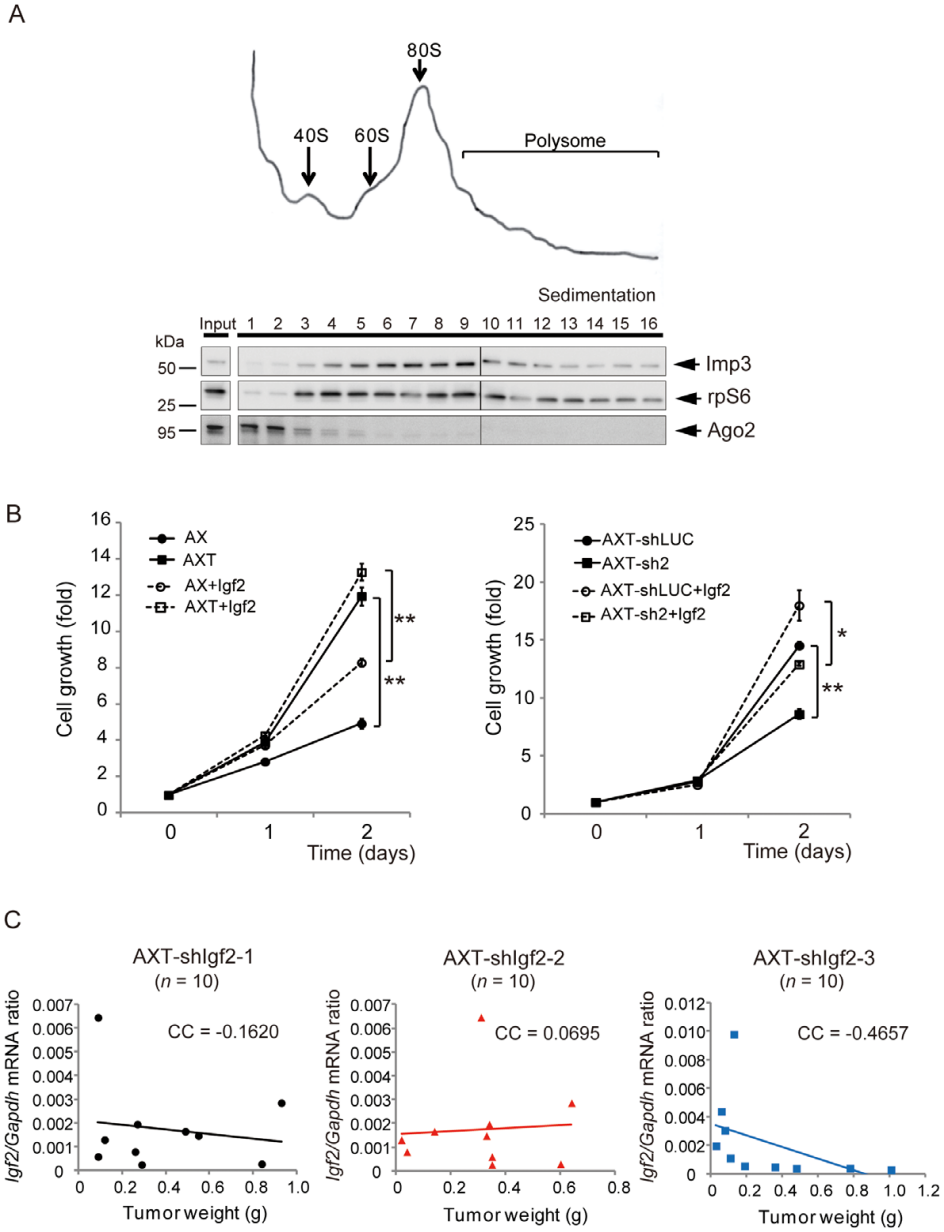
Imp3 regulates tumorigenic activity independently of Igf2 in AXT cells. (**A**) AXT cell lysate was fractionated by centrifugation on 5 to 30% sucrose gradient. The resulting absorbance profile of the gradient was determined at 254 nm for identification of ribosomal subunits, individual ribosomes, and polyribosomes (upper panel). The gradient fractions as well as the original lysate sample (Input) were subjected to immunoblot analysis with antibodies to Imp3, rpS6, and Ago2. (**B**) Cell proliferation assays for AX and AXT cells and for AXT-shLUC and AXT-sh2 cells performed under nonadherent culture conditions and in complete medium supplemented (or not) with Igf2 (50 ng/ml). *P<0.05, **P<0.001. (**C**) AXT cells expressing three different Igf2 shRNAs (AXT-shIgf2-1 to -3) were injected subcutaneously into syngeneic mice. The tumors were weighed and assayed for *Igf2* expression by real-time PCR analysis. The correlation coefficient (CC) for the two variables was determined.

Possessing six RNA binding motifs, including two RNA recognition motifs (RRMs) and four KH domains, Imp3 is implicated in the regulation of target mRNAs [7], [9], [18]–[20]. To examine the role of Imp3 in translational regulation [8] in AXT cells, we further determined its intracellular localization by centrifugation of cell lysates on a sucrose gradient. Immunoblot analysis revealed that ribosomal protein S6 (rpS6) was present in fractions 3 to 7, corresponding to ribosome subunits as well as individual ribosomes, and in fractions 8 to 16, corresponding to polysomes (Figure 6A). Imp3 was found to colocalize largely with rpS6 in the gradient fractions. In contrast, Ago2 was present mostly in fractions 1 to 3, consistent with previous observations [21]. The distribution pattern for Imp3 suggested that the protein localizes to ribosomes and polysomes.

*Igf2* mRNA has been implicated as a main target of Imp3, and activation of *Igf2* mRNA translation driven by Imp3 was previously found resulting in modulation of cellular functions such as proliferation and tumorigenic activity [18], [22]–[24] as well as tumor cell invasion [19], [24], [25]. We therefore examined whether the effects of Imp3 on cell behavior observed in this study might be mediated by Igf2. We first evaluated whether exogenous Igf2 might recapitulate the enhancement of cell proliferation induced by Imp3. The rate of proliferation of AX and Imp3-depleted AXT (AXT-sh2) cells under non-adherent conditions was increased by Igf2 in a concentration-dependent manner, with this effect reaching a plateau at concentration of 50 ng/ml in AX cells (Figure S2). However, Igf2 failed to increase the proliferation rate of AX cells to the level apparent for AXT cells. In addition, the proliferation of AXT cells was further stimulated by Igf2 (Figure 6B).

To evaluate further whether Igf2 functions downstream of Imp3 in AXT cells, we examined the tumorigenic activity of AXT cells which depleted of endogenous Igf2 by three different shRNAs (designated AXT-shIgf2 cells). Of note, the amount of *Igf2* mRNA in AXT cells was found to be very low, and the protein was undetectable (<1.5 ng/ml) in corresponding culture supernatants or tumor homogenates by ELISA [26] (data not shown). Expression of *Igf2* was attenuated in AXT-shIgf2 cells, whereas *Imp3* expression was unaffected (Figure S3). All mice injected subcutaneously with AXT-shIgf2 cells developed osteosarcoma tumors, and *Igf2* expression in these tumors did not correlate with tumor weight (Figure 6C).

Although we cannot rule out the possibility that Imp3 activates the translation of *Igf2* mRNA and that this action contributes to the phenotypic changes observed in AXT cells relative to AX cells, our results collectively suggest that the phenotypic effects of Imp3 are not attributable solely to the augmentation of Igf2 signaling.

## Discussion

Cancer cells acquire various malignant properties during the disease course. The aim of this study was to identify molecules that contribute to such changes in cancer cells with the use of two newly established osteosarcoma cell lines, AX and AXT. AXT cells, which were isolated from osteosarcoma tumors formed by AX cells, manifest a tumorigenic activity in vivo greater than that of AX cells. We identified Imp3 as a key molecule that contributes to the acquisition of malignant properties by AX cells and their associated conversion into AXT cells.

The up-regulation of Imp3 expression in tumors formed by AX cells in vivo was found not to be attributable simply to the expansion of the small population of cells that initially expresses Imp3 at high level, but was instead due to the induction of Imp3 expression during tumorigenesis. This result has important implications with regard to the evaluation of cellular tumorigenicity and the notion of cancer stem cells, in that it shows that the properties of cancer cells can change markedly in vivo.

The molecular mechanisms of re-emergence of oncofetal proteins in cancer cells remain to be fully elucidated. Our findings suggest that the up-regulation of *Imp3* expression during osteosarcoma formations could be partially attributable to epigenitic modifications (Figure S1). Previous reports indicated that IMP3 expression could be regulated by growth factor signaling in breast cancer cells [27] or miRNA in Drosophila [28]. Treatment of epigenetic modification drugs with AX cells could not fully recapitulate the high expression level of *Imp3* in AXT cells (Figure 1C and Figure S1), therefore, other upstream mechanisms could be involved during tumorigenesis in AX cells. In contrast, Ink4a/Arf knockout stromal cells, which are parental cells for AX cells, did not exhibit as much response to the epigenetic modifiers as AX cells (data not shown), which might reflect the differential plasticity in epigenetic regulation between normal cells and cancer cells [29].

Both gain and loss of function of Imp3 in osteosarcoma cells revealed that Imp3 confers the ability to undergo anchorage-independent growth, loss of contact inhibition, and resistance to anoikis in vitro, all of which contribute to the development of tumorgenic potential. Previous studies reported that Imp3 enhances cell proliferation and invasion [14], [18], [22], [24], [25], [27]. Collectively Imp3 might contribute to the regulation of molecules involved in cell cycle and remodeling of cytoskeleton.

We found that Imp3 was associated with individual ribosomes, ribosome subunits, and polysomes in AXT cells, consistent with the proposed role for Imp3 in the regulation of translation [7], [8], [18]. The oncogenic effects of Imp3 have been suggested to be mediated through Igf2, the mRNA for which is translationally activated by Imp3 [18], [22]–[24]. However, our findings indicate that the malignant properties conferred by Imp3 are not attributable to the action of Igf2 alone. The *Igf2* expression in AXT cells was thus found to be extremely low, and the encoded protein in tumors was not detectable with ELISA. Furthermore, exogenous Igf2 did not provide growth advantage for AX cells as great as that conferred by Imp3 expression, and shRNA-mediated suppression of endogenous *Igf2* expression did not affect the tumorigenic activity of AXT cells. Translational regulation of several molecules such as CD44, CD164, and MMP9 has been suggested to underlie changes in cellular phenotype induced by Imp3, without being accompanied by modification of Igf2 [25], [27]. Collectively, our results suggest that deregulation of Imp3 expression in AXT cells might affect the expression of key molecules other than Igf2, as has been suggested previously [20].

We found that knockdown of Imp3 in AXT cells resulted in a marked reduction in tumorigenic activity in vivo. Moreover, 90% of the human osteosarcoma specimens analyzed were positive for Imp3 expression. IMP3 has previously been suggested as a prognostic marker for metastatic or angiogenic potential in human osteosarcoma [30], [31]. Our results implicate Imp3 as a molecule capable of conferring critical properties to transformed cells for tumorigenic ability in vivo, which is indispensable for tumor-initiating cells, often consistent with cancer stem cells [32], [33]. Thus Imp3 might be a key regulator of cancer stem-like characteristics in cancer cells, in which case it may also be a potential therapeutic target for osteosarcoma as well as other tumor types.

## Supporting Information

Figure S1 **Effects of epigenetic modifiers on Imp3 expression.** Real-time PCR analysis of Imp3 expression in AX cells after treatment with DNMT1 inhibitor; 5AzaD and HDAC inhibitors; TSA, VPA or SAHA at the indicated concentration. *P<0.05, **P<0.01, ***P<0.001. NS, not significant.
(TIF)

Figure S2 **Effect of Igf2 on osteosarcoma cell proliferation in vitro.** The proliferation of AX and AXT-sh2 cells was assayed under nonadherent culture conditions supplemented with the indicated concentrations (0 to 500 ng/ml) of Igf2.
(TIF)

Figure S3 **Depletion of Igf2 mRNA in AXT cells.** The expression levels of Igf2 and Imp3 in AXT-shIgf2 cells were evaluated by real-time PCR analysis.
(TIF)

Table S1 **Sequences of PCR primers, predicted PCR product sizes, and target sequences for shRNAs.**
(DOCX)

Table S2 **Genes whose expression is up-regulated in AXT cells compared with AX cells.**
(DOCX)

Table S3 **Knockdown of Imp3 in AXT cells suppresses tumorigenic activity in vivo.**
(DOCX)
