# Supplementary figures and images for: Up-Regulation of Imp3 Confers In Vivo Tumorigenicity on Murine Osteosarcoma Cells
Source: PLoS One. 2012 Nov 30;7(11):e50621. doi: 10.1371/journal.pone.0050621 (PMC3511546; doi:10.1371/journal.pone.0050621)

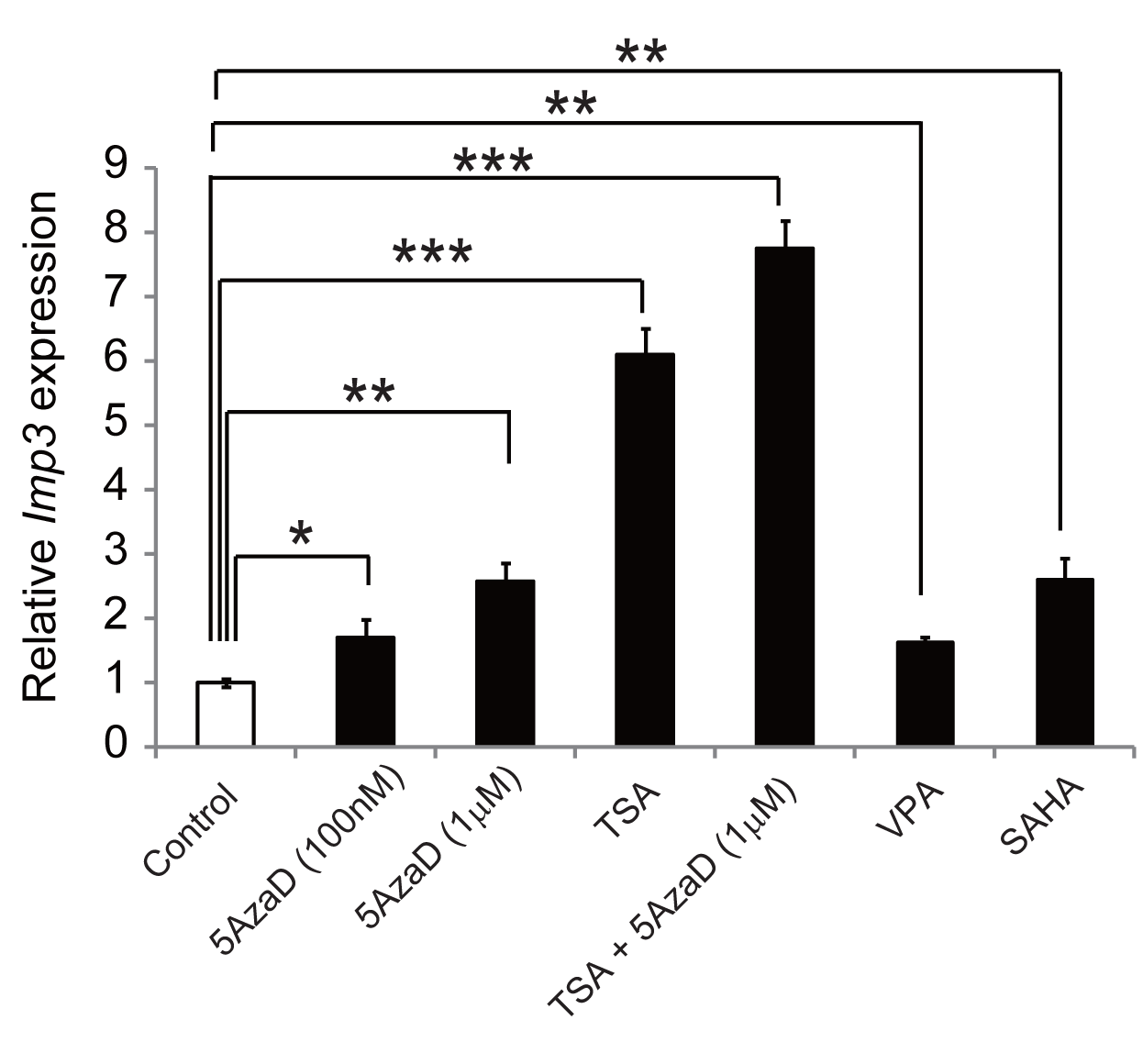

Supplement: Figure S1 — Effects of epigenetic modifiers on Imp3 expression. Real-time PCR analysis of Imp3 expression in AX cells after treatment with DNMT1 inhibitor; 5AzaD and HDAC inhibitors; TSA, VPA or SAHA at the indicated concentration. *P<0.05, **P<0.01, ***P<0.001. NS, not significant. (TIF) [file pone.0050621.s001.tif]

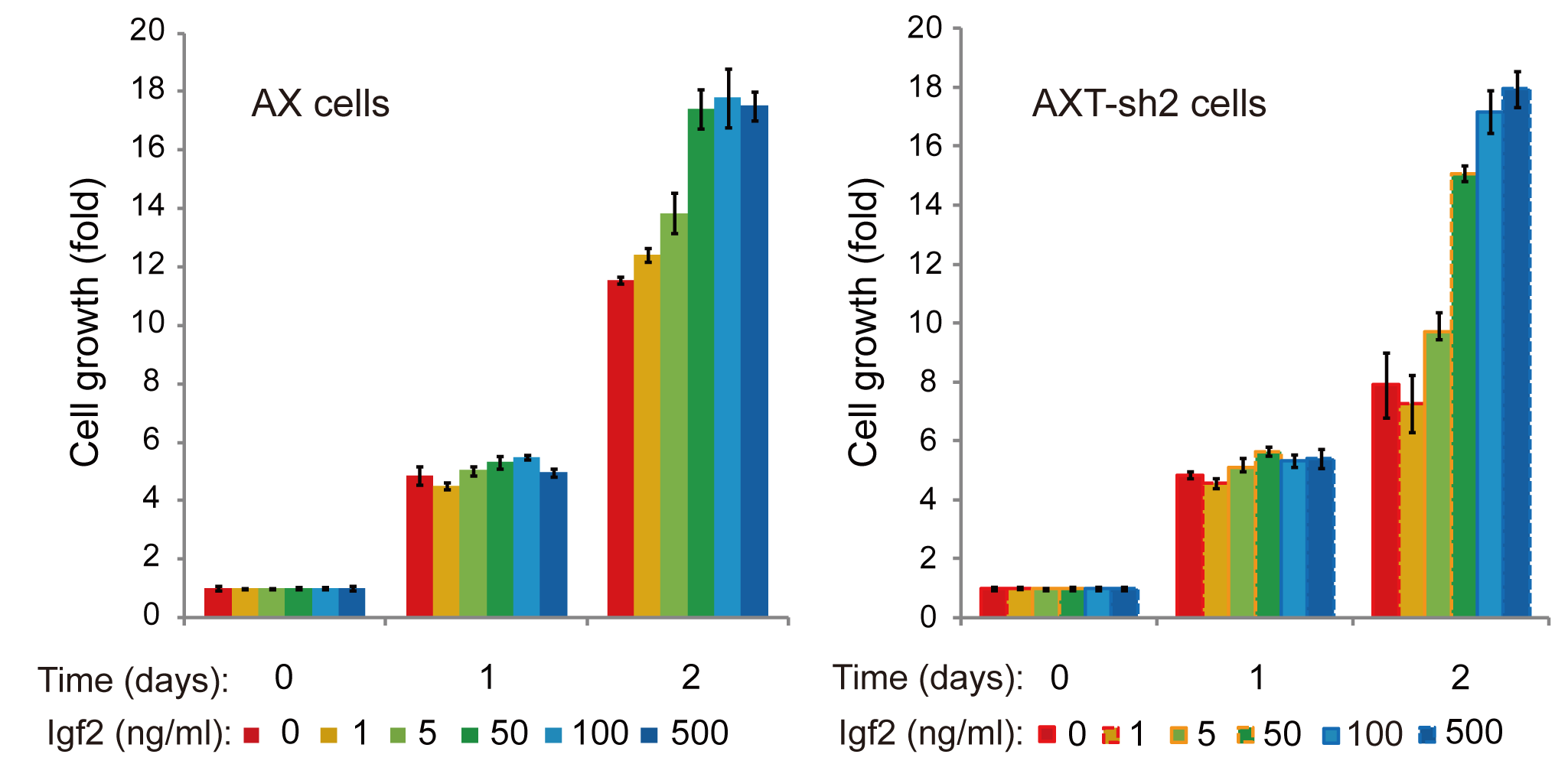

Supplement: Figure S2 — Effect of Igf2 on osteosarcoma cell proliferation in vitro. The proliferation of AX and AXT-sh2 cells was assayed under nonadherent culture conditions supplemented with the indicated concentrations (0 to 500 ng/ml) of Igf2. (TIF) [file pone.0050621.s002.tif]

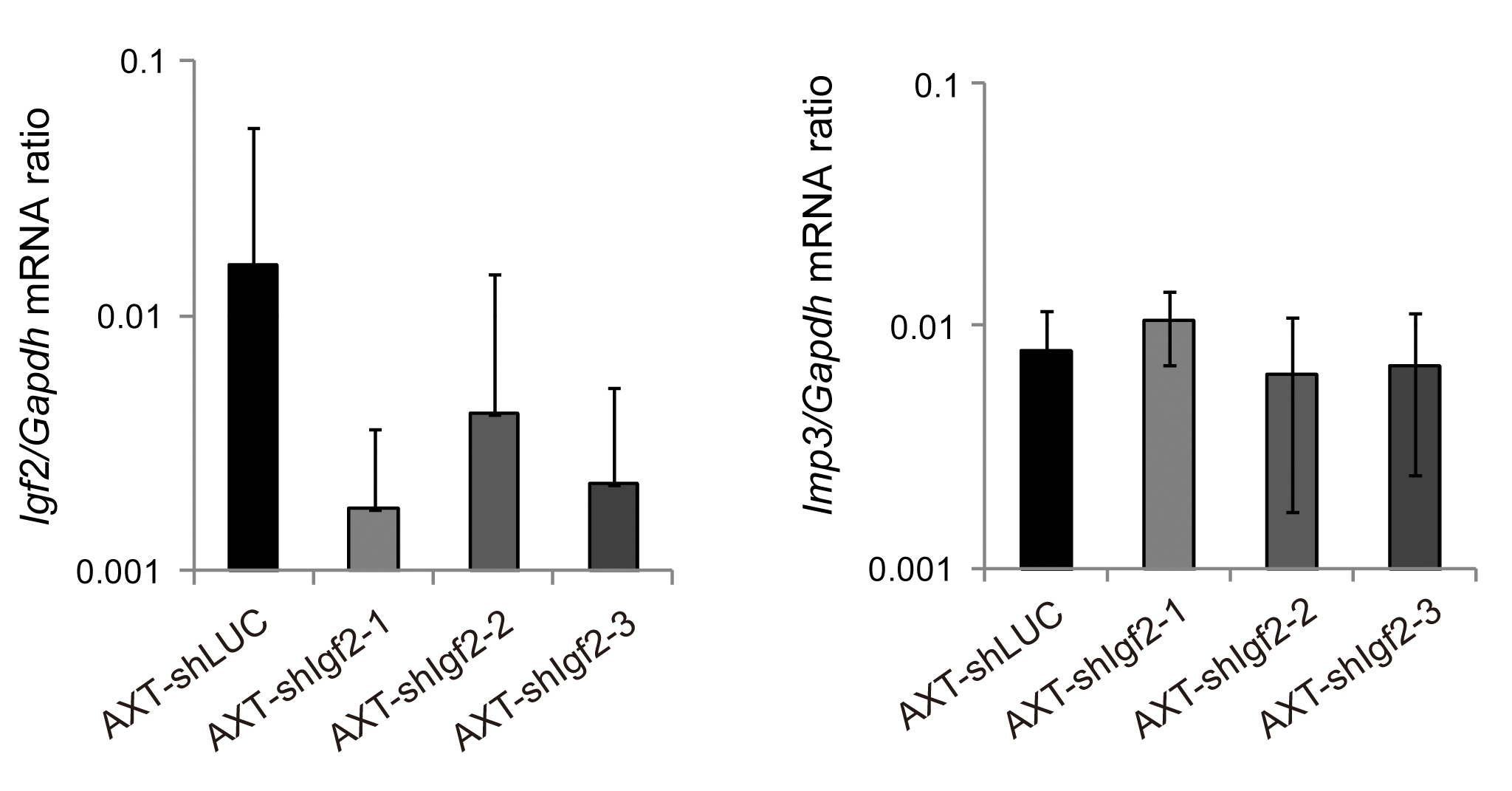

Supplement: Figure S3 — Depletion of Igf2 mRNA in AXT cells. The expression levels of Igf2 and Imp3 in AXT-shIgf2 cells were evaluated by real-time PCR analysis. (TIF) [file pone.0050621.s003.tif]
