# Supplementary material for: Up-Regulation of Imp3 Confers In Vivo Tumorigenicity on Murine Osteosarcoma Cells
Source: PLoS One. 2012 Nov 30;7(11):e50621. doi: 10.1371/journal.pone.0050621 (PMC3511546; doi:10.1371/journal.pone.0050621)
Supplement: Table S1 — Sequences of PCR primers, predicted PCR product sizes, and target sequences for shRNAs. (DOCX) [file pone.0050621.s004.docx]

**Table S1. Sequences of PCR primers, predicted PCR product sizes, and target sequences for shRNAs.**

| [Gene](C:\\Users\\arisa\\Desktop\\Imp3論文用\\フィギュアfinal\\Supple.Table.S1.xlsx#Sheet1!B4) | [Forword primer (5’→3’)](C:\\Users\\arisa\\Desktop\\Imp3論文用\\フィギュアfinal\\Supple.Table.S1.xlsx#Sheet1!B4) | [Reverse primer (5’→3’)](C:\\Users\\arisa\\Desktop\\Imp3論文用\\フィギュアfinal\\Supple.Table.S1.xlsx#Sheet1!B4) | [Product Size (bp)](C:\\Users\\arisa\\Desktop\\Imp3論文用\\フィギュアfinal\\Supple.Table.S1.xlsx#Sheet1!B4) |
| --- | --- | --- | --- |
| *[Gapdh](C:\\Users\\arisa\\Desktop\\Imp3論文用\\フィギュアfinal\\Supple.Table.S1.xlsx#Sheet1!B4)* | [GTGAAGGTCGGTGTGAACG](C:\\Users\\arisa\\Desktop\\Imp3論文用\\フィギュアfinal\\Supple.Table.S1.xlsx#Sheet1!B4) | [GACCATGTAGTTGAGGTCAATG](C:\\Users\\arisa\\Desktop\\Imp3論文用\\フィギュアfinal\\Supple.Table.S1.xlsx#Sheet1!B4) | [123](C:\\Users\\arisa\\Desktop\\Imp3論文用\\フィギュアfinal\\Supple.Table.S1.xlsx#Sheet1!B4) |
| *[Imp3](C:\\Users\\arisa\\Desktop\\Imp3論文用\\フィギュアfinal\\Supple.Table.S1.xlsx#Sheet1!B4)* | [CCCAGTTTGTTGGAGCCATT](C:\\Users\\arisa\\Desktop\\Imp3論文用\\フィギュアfinal\\Supple.Table.S1.xlsx#Sheet1!B4) | [CCTGTATTCTCCTTACGATG](C:\\Users\\arisa\\Desktop\\Imp3論文用\\フィギュアfinal\\Supple.Table.S1.xlsx#Sheet1!B4) | [103](C:\\Users\\arisa\\Desktop\\Imp3論文用\\フィギュアfinal\\Supple.Table.S1.xlsx#Sheet1!B4) |
| *[GFP](C:\\Users\\arisa\\Desktop\\Imp3論文用\\フィギュアfinal\\Supple.Table.S1.xlsx#Sheet1!B4)* | [GACGTAAACGGCCACAAGTT](C:\\Users\\arisa\\Desktop\\Imp3論文用\\フィギュアfinal\\Supple.Table.S1.xlsx#Sheet1!B4) | [TTGCCGGTGGTGCAGATGAA](C:\\Users\\arisa\\Desktop\\Imp3論文用\\フィギュアfinal\\Supple.Table.S1.xlsx#Sheet1!B4) | [95](C:\\Users\\arisa\\Desktop\\Imp3論文用\\フィギュアfinal\\Supple.Table.S1.xlsx#Sheet1!B4) |
| *[Igf2](C:\\Users\\arisa\\Desktop\\Imp3論文用\\フィギュアfinal\\Supple.Table.S1.xlsx#Sheet1!B4)* | [TCAGTTTGTCTGTTCGGACC](C:\\Users\\arisa\\Desktop\\Imp3論文用\\フィギュアfinal\\Supple.Table.S1.xlsx#Sheet1!B4) | [AAGCAGCACTCTTCCACGAT](C:\\Users\\arisa\\Desktop\\Imp3論文用\\フィギュアfinal\\Supple.Table.S1.xlsx#Sheet1!B4) | [93](C:\\Users\\arisa\\Desktop\\Imp3論文用\\フィギュアfinal\\Supple.Table.S1.xlsx#Sheet1!B4) |

| [shRNA](C:\\Users\\arisa\\Desktop\\Imp3論文用\\フィギュアfinal\\Supple.Table.S1.xlsx#Sheet1!B10) | [sense oligonucleotides (5’→3’)](C:\\Users\\arisa\\Desktop\\Imp3論文用\\フィギュアfinal\\Supple.Table.S1.xlsx#Sheet1!B10) |
| --- | --- |
| [Luciferase (LUC)](C:\\Users\\arisa\\Desktop\\Imp3論文用\\フィギュアfinal\\Supple.Table.S1.xlsx#Sheet1!B10) | [CGTACGCGGAATACTTCGA](C:\\Users\\arisa\\Desktop\\Imp3論文用\\フィギュアfinal\\Supple.Table.S1.xlsx#Sheet1!B10) |
| [Imp3 shRNA1](C:\\Users\\arisa\\Desktop\\Imp3論文用\\フィギュアfinal\\Supple.Table.S1.xlsx#Sheet1!B10) | [GGATTCGTAAACTTCAGAT](C:\\Users\\arisa\\Desktop\\Imp3論文用\\フィギュアfinal\\Supple.Table.S1.xlsx#Sheet1!B10) |
| [Imp3 shRNA2](C:\\Users\\arisa\\Desktop\\Imp3論文用\\フィギュアfinal\\Supple.Table.S1.xlsx#Sheet1!B10) | [GACAAGCCTTAGACAAACT](C:\\Users\\arisa\\Desktop\\Imp3論文用\\フィギュアfinal\\Supple.Table.S1.xlsx#Sheet1!B10) |
| [IGF2 shRNA1](C:\\Users\\arisa\\Desktop\\Imp3論文用\\フィギュアfinal\\Supple.Table.S1.xlsx#Sheet1!B10) | [GGGCAAGTTCTTCCAATAT](C:\\Users\\arisa\\Desktop\\Imp3論文用\\フィギュアfinal\\Supple.Table.S1.xlsx#Sheet1!B10) |
| [IGF2 shRNA2](C:\\Users\\arisa\\Desktop\\Imp3論文用\\フィギュアfinal\\Supple.Table.S1.xlsx#Sheet1!B10) | [GCTTGCCAAAGAGCTCAAA](C:\\Users\\arisa\\Desktop\\Imp3論文用\\フィギュアfinal\\Supple.Table.S1.xlsx#Sheet1!B10) |
| [IGF2 shRNA3](C:\\Users\\arisa\\Desktop\\Imp3論文用\\フィギュアfinal\\Supple.Table.S1.xlsx#Sheet1!B10) | [GGAGATGTCCAGCAACCAT](C:\\Users\\arisa\\Desktop\\Imp3論文用\\フィギュアfinal\\Supple.Table.S1.xlsx#Sheet1!B10) |
