# Supplementary material for: Up-Regulation of Imp3 Confers In Vivo Tumorigenicity on Murine Osteosarcoma Cells
Source: PLoS One. 2012 Nov 30;7(11):e50621. doi: 10.1371/journal.pone.0050621 (PMC3511546; doi:10.1371/journal.pone.0050621)
Supplement: Table S2 — Genes whose expression is up-regulated in AXT cells compared with AX cells. (DOCX) [file pone.0050621.s005.docx]

**Table S2. Genes whose expression is up-regulated in AXT cells compared with AX cells.**

| [Gene](C:\\Users\\arisa\\Desktop\\Imp3論文用\\フィギュアfinal\\Supple.Table.S2.xlsx#Sup.Table1!B4) | [Normalized expression level](C:\\Users\\arisa\\Desktop\\Imp3論文用\\フィギュアfinal\\Supple.Table.S2.xlsx#Sup.Table1!B4) | | |
| --- | --- | --- | --- |
|  | [AXT](C:\\Users\\arisa\\Desktop\\Imp3論文用\\フィギュアfinal\\Supple.Table.S2.xlsx#Sup.Table1!B4) | [AX](C:\\Users\\arisa\\Desktop\\Imp3論文用\\フィギュアfinal\\Supple.Table.S2.xlsx#Sup.Table1!B4) | [log](C:\\Users\\arisa\\Desktop\\Imp3論文用\\フィギュアfinal\\Supple.Table.S2.xlsx#Sup.Table1!B4)_[2](C:\\Users\\arisa\\Desktop\\Imp3論文用\\フィギュアfinal\\Supple.Table.S2.xlsx#Sup.Table1!B4)_ [(AXT/AX)](C:\\Users\\arisa\\Desktop\\Imp3論文用\\フィギュアfinal\\Supple.Table.S2.xlsx#Sup.Table1!B4) |
| *[4930517K11Rik](C:\\Users\\arisa\\Desktop\\Imp3論文用\\フィギュアfinal\\Supple.Table.S2.xlsx#Sup.Table1!B4)* | [6262.6](C:\\Users\\arisa\\Desktop\\Imp3論文用\\フィギュアfinal\\Supple.Table.S2.xlsx#Sup.Table1!B4) | [23.2](C:\\Users\\arisa\\Desktop\\Imp3論文用\\フィギュアfinal\\Supple.Table.S2.xlsx#Sup.Table1!B4) | [8.1](C:\\Users\\arisa\\Desktop\\Imp3論文用\\フィギュアfinal\\Supple.Table.S2.xlsx#Sup.Table1!B4) |
| *[Dync1i1](C:\\Users\\arisa\\Desktop\\Imp3論文用\\フィギュアfinal\\Supple.Table.S2.xlsx#Sup.Table1!B4)* | [588.7](C:\\Users\\arisa\\Desktop\\Imp3論文用\\フィギュアfinal\\Supple.Table.S2.xlsx#Sup.Table1!B4) | [6.4](C:\\Users\\arisa\\Desktop\\Imp3論文用\\フィギュアfinal\\Supple.Table.S2.xlsx#Sup.Table1!B4) | [6.5](C:\\Users\\arisa\\Desktop\\Imp3論文用\\フィギュアfinal\\Supple.Table.S2.xlsx#Sup.Table1!B4) |
| *[Khk](C:\\Users\\arisa\\Desktop\\Imp3論文用\\フィギュアfinal\\Supple.Table.S2.xlsx#Sup.Table1!B4)* | [708.6](C:\\Users\\arisa\\Desktop\\Imp3論文用\\フィギュアfinal\\Supple.Table.S2.xlsx#Sup.Table1!B4) | [10.5](C:\\Users\\arisa\\Desktop\\Imp3論文用\\フィギュアfinal\\Supple.Table.S2.xlsx#Sup.Table1!B4) | [6.1](C:\\Users\\arisa\\Desktop\\Imp3論文用\\フィギュアfinal\\Supple.Table.S2.xlsx#Sup.Table1!B4) |
| *[Piwil2](C:\\Users\\arisa\\Desktop\\Imp3論文用\\フィギュアfinal\\Supple.Table.S2.xlsx#Sup.Table1!B4)* | [568.0](C:\\Users\\arisa\\Desktop\\Imp3論文用\\フィギュアfinal\\Supple.Table.S2.xlsx#Sup.Table1!B4) | [8.5](C:\\Users\\arisa\\Desktop\\Imp3論文用\\フィギュアfinal\\Supple.Table.S2.xlsx#Sup.Table1!B4) | [6.1](C:\\Users\\arisa\\Desktop\\Imp3論文用\\フィギュアfinal\\Supple.Table.S2.xlsx#Sup.Table1!B4) |
| *[Armcx2](C:\\Users\\arisa\\Desktop\\Imp3論文用\\フィギュアfinal\\Supple.Table.S2.xlsx#Sup.Table1!B4)* | [597.5](C:\\Users\\arisa\\Desktop\\Imp3論文用\\フィギュアfinal\\Supple.Table.S2.xlsx#Sup.Table1!B4) | [9.6](C:\\Users\\arisa\\Desktop\\Imp3論文用\\フィギュアfinal\\Supple.Table.S2.xlsx#Sup.Table1!B4) | [6.0](C:\\Users\\arisa\\Desktop\\Imp3論文用\\フィギュアfinal\\Supple.Table.S2.xlsx#Sup.Table1!B4) |
| *[Xlr3b](C:\\Users\\arisa\\Desktop\\Imp3論文用\\フィギュアfinal\\Supple.Table.S2.xlsx#Sup.Table1!B4)* | [494.0](C:\\Users\\arisa\\Desktop\\Imp3論文用\\フィギュアfinal\\Supple.Table.S2.xlsx#Sup.Table1!B4) | [9.3](C:\\Users\\arisa\\Desktop\\Imp3論文用\\フィギュアfinal\\Supple.Table.S2.xlsx#Sup.Table1!B4) | [5.7](C:\\Users\\arisa\\Desktop\\Imp3論文用\\フィギュアfinal\\Supple.Table.S2.xlsx#Sup.Table1!B4) |
| *[Itm2a](C:\\Users\\arisa\\Desktop\\Imp3論文用\\フィギュアfinal\\Supple.Table.S2.xlsx#Sup.Table1!B4)* | [742.0](C:\\Users\\arisa\\Desktop\\Imp3論文用\\フィギュアfinal\\Supple.Table.S2.xlsx#Sup.Table1!B4) | [17.8](C:\\Users\\arisa\\Desktop\\Imp3論文用\\フィギュアfinal\\Supple.Table.S2.xlsx#Sup.Table1!B4) | [5.4](C:\\Users\\arisa\\Desktop\\Imp3論文用\\フィギュアfinal\\Supple.Table.S2.xlsx#Sup.Table1!B4) |
| *[3830403N18Rik](C:\\Users\\arisa\\Desktop\\Imp3論文用\\フィギュアfinal\\Supple.Table.S2.xlsx#Sup.Table1!B4)* | [270.2](C:\\Users\\arisa\\Desktop\\Imp3論文用\\フィギュアfinal\\Supple.Table.S2.xlsx#Sup.Table1!B4) | [9.9](C:\\Users\\arisa\\Desktop\\Imp3論文用\\フィギュアfinal\\Supple.Table.S2.xlsx#Sup.Table1!B4) | [4.8](C:\\Users\\arisa\\Desktop\\Imp3論文用\\フィギュアfinal\\Supple.Table.S2.xlsx#Sup.Table1!B4) |
| *[Ldhb](C:\\Users\\arisa\\Desktop\\Imp3論文用\\フィギュアfinal\\Supple.Table.S2.xlsx#Sup.Table1!B4)* | [254.7](C:\\Users\\arisa\\Desktop\\Imp3論文用\\フィギュアfinal\\Supple.Table.S2.xlsx#Sup.Table1!B4) | [12.5](C:\\Users\\arisa\\Desktop\\Imp3論文用\\フィギュアfinal\\Supple.Table.S2.xlsx#Sup.Table1!B4) | [4.3](C:\\Users\\arisa\\Desktop\\Imp3論文用\\フィギュアfinal\\Supple.Table.S2.xlsx#Sup.Table1!B4) |
| *[Xlr3a](C:\\Users\\arisa\\Desktop\\Imp3論文用\\フィギュアfinal\\Supple.Table.S2.xlsx#Sup.Table1!B4)* | [253.2](C:\\Users\\arisa\\Desktop\\Imp3論文用\\フィギュアfinal\\Supple.Table.S2.xlsx#Sup.Table1!B4) | [13.4](C:\\Users\\arisa\\Desktop\\Imp3論文用\\フィギュアfinal\\Supple.Table.S2.xlsx#Sup.Table1!B4) | [4.2](C:\\Users\\arisa\\Desktop\\Imp3論文用\\フィギュアfinal\\Supple.Table.S2.xlsx#Sup.Table1!B4) |
| *[Scara3](C:\\Users\\arisa\\Desktop\\Imp3論文用\\フィギュアfinal\\Supple.Table.S2.xlsx#Sup.Table1!B4)* | [144.4](C:\\Users\\arisa\\Desktop\\Imp3論文用\\フィギュアfinal\\Supple.Table.S2.xlsx#Sup.Table1!B4) | [7.8](C:\\Users\\arisa\\Desktop\\Imp3論文用\\フィギュアfinal\\Supple.Table.S2.xlsx#Sup.Table1!B4) | [4.2](C:\\Users\\arisa\\Desktop\\Imp3論文用\\フィギュアfinal\\Supple.Table.S2.xlsx#Sup.Table1!B4) |
| *[Gm266](C:\\Users\\arisa\\Desktop\\Imp3論文用\\フィギュアfinal\\Supple.Table.S2.xlsx#Sup.Table1!B4)* | [96.3](C:\\Users\\arisa\\Desktop\\Imp3論文用\\フィギュアfinal\\Supple.Table.S2.xlsx#Sup.Table1!B4) | [5.6](C:\\Users\\arisa\\Desktop\\Imp3論文用\\フィギュアfinal\\Supple.Table.S2.xlsx#Sup.Table1!B4) | [4.1](C:\\Users\\arisa\\Desktop\\Imp3論文用\\フィギュアfinal\\Supple.Table.S2.xlsx#Sup.Table1!B4) |
| *[Sema3a](C:\\Users\\arisa\\Desktop\\Imp3論文用\\フィギュアfinal\\Supple.Table.S2.xlsx#Sup.Table1!B4)* | [111.3](C:\\Users\\arisa\\Desktop\\Imp3論文用\\フィギュアfinal\\Supple.Table.S2.xlsx#Sup.Table1!B4) | [6.6](C:\\Users\\arisa\\Desktop\\Imp3論文用\\フィギュアfinal\\Supple.Table.S2.xlsx#Sup.Table1!B4) | [4.1](C:\\Users\\arisa\\Desktop\\Imp3論文用\\フィギュアfinal\\Supple.Table.S2.xlsx#Sup.Table1!B4) |
| *[Pcbd1](C:\\Users\\arisa\\Desktop\\Imp3論文用\\フィギュアfinal\\Supple.Table.S2.xlsx#Sup.Table1!B4)* | [68.1](C:\\Users\\arisa\\Desktop\\Imp3論文用\\フィギュアfinal\\Supple.Table.S2.xlsx#Sup.Table1!B4) | [4.3](C:\\Users\\arisa\\Desktop\\Imp3論文用\\フィギュアfinal\\Supple.Table.S2.xlsx#Sup.Table1!B4) | [4.0](C:\\Users\\arisa\\Desktop\\Imp3論文用\\フィギュアfinal\\Supple.Table.S2.xlsx#Sup.Table1!B4) |
| *[Hfe](C:\\Users\\arisa\\Desktop\\Imp3論文用\\フィギュアfinal\\Supple.Table.S2.xlsx#Sup.Table1!B4)* | [79.9](C:\\Users\\arisa\\Desktop\\Imp3論文用\\フィギュアfinal\\Supple.Table.S2.xlsx#Sup.Table1!B4) | [5.3](C:\\Users\\arisa\\Desktop\\Imp3論文用\\フィギュアfinal\\Supple.Table.S2.xlsx#Sup.Table1!B4) | [3.9](C:\\Users\\arisa\\Desktop\\Imp3論文用\\フィギュアfinal\\Supple.Table.S2.xlsx#Sup.Table1!B4) |
| *[Cthrc1](C:\\Users\\arisa\\Desktop\\Imp3論文用\\フィギュアfinal\\Supple.Table.S2.xlsx#Sup.Table1!B4)* | [63.5](C:\\Users\\arisa\\Desktop\\Imp3論文用\\フィギュアfinal\\Supple.Table.S2.xlsx#Sup.Table1!B4) | [4.3](C:\\Users\\arisa\\Desktop\\Imp3論文用\\フィギュアfinal\\Supple.Table.S2.xlsx#Sup.Table1!B4) | [3.9](C:\\Users\\arisa\\Desktop\\Imp3論文用\\フィギュアfinal\\Supple.Table.S2.xlsx#Sup.Table1!B4) |
| *[Cbr3](C:\\Users\\arisa\\Desktop\\Imp3論文用\\フィギュアfinal\\Supple.Table.S2.xlsx#Sup.Table1!B4)* | [281.1](C:\\Users\\arisa\\Desktop\\Imp3論文用\\フィギュアfinal\\Supple.Table.S2.xlsx#Sup.Table1!B4) | [20.2](C:\\Users\\arisa\\Desktop\\Imp3論文用\\フィギュアfinal\\Supple.Table.S2.xlsx#Sup.Table1!B4) | [3.8](C:\\Users\\arisa\\Desktop\\Imp3論文用\\フィギュアfinal\\Supple.Table.S2.xlsx#Sup.Table1!B4) |
| *[Igf2bp3](C:\\Users\\arisa\\Desktop\\Imp3論文用\\フィギュアfinal\\Supple.Table.S2.xlsx#Sup.Table1!B4)* | [153.2](C:\\Users\\arisa\\Desktop\\Imp3論文用\\フィギュアfinal\\Supple.Table.S2.xlsx#Sup.Table1!B4) | [11.1](C:\\Users\\arisa\\Desktop\\Imp3論文用\\フィギュアfinal\\Supple.Table.S2.xlsx#Sup.Table1!B4) | [3.8](C:\\Users\\arisa\\Desktop\\Imp3論文用\\フィギュアfinal\\Supple.Table.S2.xlsx#Sup.Table1!B4) |
| *[Socs2](C:\\Users\\arisa\\Desktop\\Imp3論文用\\フィギュアfinal\\Supple.Table.S2.xlsx#Sup.Table1!B4)* | [105.0](C:\\Users\\arisa\\Desktop\\Imp3論文用\\フィギュアfinal\\Supple.Table.S2.xlsx#Sup.Table1!B4) | [7.7](C:\\Users\\arisa\\Desktop\\Imp3論文用\\フィギュアfinal\\Supple.Table.S2.xlsx#Sup.Table1!B4) | [3.8](C:\\Users\\arisa\\Desktop\\Imp3論文用\\フィギュアfinal\\Supple.Table.S2.xlsx#Sup.Table1!B4) |
| *[F2r](C:\\Users\\arisa\\Desktop\\Imp3論文用\\フィギュアfinal\\Supple.Table.S2.xlsx#Sup.Table1!B4)* | [184.5](C:\\Users\\arisa\\Desktop\\Imp3論文用\\フィギュアfinal\\Supple.Table.S2.xlsx#Sup.Table1!B4) | [14.3](C:\\Users\\arisa\\Desktop\\Imp3論文用\\フィギュアfinal\\Supple.Table.S2.xlsx#Sup.Table1!B4) | [3.7](C:\\Users\\arisa\\Desktop\\Imp3論文用\\フィギュアfinal\\Supple.Table.S2.xlsx#Sup.Table1!B4) |
| *[Egfl7](C:\\Users\\arisa\\Desktop\\Imp3論文用\\フィギュアfinal\\Supple.Table.S2.xlsx#Sup.Table1!B4)* | [324.2](C:\\Users\\arisa\\Desktop\\Imp3論文用\\フィギュアfinal\\Supple.Table.S2.xlsx#Sup.Table1!B4) | [25.7](C:\\Users\\arisa\\Desktop\\Imp3論文用\\フィギュアfinal\\Supple.Table.S2.xlsx#Sup.Table1!B4) | [3.7](C:\\Users\\arisa\\Desktop\\Imp3論文用\\フィギュアfinal\\Supple.Table.S2.xlsx#Sup.Table1!B4) |
| *[Car14](C:\\Users\\arisa\\Desktop\\Imp3論文用\\フィギュアfinal\\Supple.Table.S2.xlsx#Sup.Table1!B4)* | [81.5](C:\\Users\\arisa\\Desktop\\Imp3論文用\\フィギュアfinal\\Supple.Table.S2.xlsx#Sup.Table1!B4) | [7.7](C:\\Users\\arisa\\Desktop\\Imp3論文用\\フィギュアfinal\\Supple.Table.S2.xlsx#Sup.Table1!B4) | [3.4](C:\\Users\\arisa\\Desktop\\Imp3論文用\\フィギュアfinal\\Supple.Table.S2.xlsx#Sup.Table1!B4) |
| *[Mpp3](C:\\Users\\arisa\\Desktop\\Imp3論文用\\フィギュアfinal\\Supple.Table.S2.xlsx#Sup.Table1!B4)* | [72.0](C:\\Users\\arisa\\Desktop\\Imp3論文用\\フィギュアfinal\\Supple.Table.S2.xlsx#Sup.Table1!B4) | [6.9](C:\\Users\\arisa\\Desktop\\Imp3論文用\\フィギュアfinal\\Supple.Table.S2.xlsx#Sup.Table1!B4) | [3.4](C:\\Users\\arisa\\Desktop\\Imp3論文用\\フィギュアfinal\\Supple.Table.S2.xlsx#Sup.Table1!B4) |
| *[Cd302](C:\\Users\\arisa\\Desktop\\Imp3論文用\\フィギュアfinal\\Supple.Table.S2.xlsx#Sup.Table1!B4)* | [101.9](C:\\Users\\arisa\\Desktop\\Imp3論文用\\フィギュアfinal\\Supple.Table.S2.xlsx#Sup.Table1!B4) | [9.8](C:\\Users\\arisa\\Desktop\\Imp3論文用\\フィギュアfinal\\Supple.Table.S2.xlsx#Sup.Table1!B4) | [3.4](C:\\Users\\arisa\\Desktop\\Imp3論文用\\フィギュアfinal\\Supple.Table.S2.xlsx#Sup.Table1!B4) |
| *[2610318N02Rik](C:\\Users\\arisa\\Desktop\\Imp3論文用\\フィギュアfinal\\Supple.Table.S2.xlsx#Sup.Table1!B4)* | [50.3](C:\\Users\\arisa\\Desktop\\Imp3論文用\\フィギュアfinal\\Supple.Table.S2.xlsx#Sup.Table1!B4) | [4.9](C:\\Users\\arisa\\Desktop\\Imp3論文用\\フィギュアfinal\\Supple.Table.S2.xlsx#Sup.Table1!B4) | [3.4](C:\\Users\\arisa\\Desktop\\Imp3論文用\\フィギュアfinal\\Supple.Table.S2.xlsx#Sup.Table1!B4) |
| *[Eps8](C:\\Users\\arisa\\Desktop\\Imp3論文用\\フィギュアfinal\\Supple.Table.S2.xlsx#Sup.Table1!B4)* | [276.9](C:\\Users\\arisa\\Desktop\\Imp3論文用\\フィギュアfinal\\Supple.Table.S2.xlsx#Sup.Table1!B4) | [27.1](C:\\Users\\arisa\\Desktop\\Imp3論文用\\フィギュアfinal\\Supple.Table.S2.xlsx#Sup.Table1!B4) | [3.4](C:\\Users\\arisa\\Desktop\\Imp3論文用\\フィギュアfinal\\Supple.Table.S2.xlsx#Sup.Table1!B4) |
| *[Ada](C:\\Users\\arisa\\Desktop\\Imp3論文用\\フィギュアfinal\\Supple.Table.S2.xlsx#Sup.Table1!B4)* | [81.2](C:\\Users\\arisa\\Desktop\\Imp3論文用\\フィギュアfinal\\Supple.Table.S2.xlsx#Sup.Table1!B4) | [8.2](C:\\Users\\arisa\\Desktop\\Imp3論文用\\フィギュアfinal\\Supple.Table.S2.xlsx#Sup.Table1!B4) | [3.3](C:\\Users\\arisa\\Desktop\\Imp3論文用\\フィギュアfinal\\Supple.Table.S2.xlsx#Sup.Table1!B4) |
| *[Ifitm1](C:\\Users\\arisa\\Desktop\\Imp3論文用\\フィギュアfinal\\Supple.Table.S2.xlsx#Sup.Table1!B4)* | [650.9](C:\\Users\\arisa\\Desktop\\Imp3論文用\\フィギュアfinal\\Supple.Table.S2.xlsx#Sup.Table1!B4) | [67.0](C:\\Users\\arisa\\Desktop\\Imp3論文用\\フィギュアfinal\\Supple.Table.S2.xlsx#Sup.Table1!B4) | [3.3](C:\\Users\\arisa\\Desktop\\Imp3論文用\\フィギュアfinal\\Supple.Table.S2.xlsx#Sup.Table1!B4) |
| *[Loxl3](C:\\Users\\arisa\\Desktop\\Imp3論文用\\フィギュアfinal\\Supple.Table.S2.xlsx#Sup.Table1!B4)* | [152.9](C:\\Users\\arisa\\Desktop\\Imp3論文用\\フィギュアfinal\\Supple.Table.S2.xlsx#Sup.Table1!B4) | [15.8](C:\\Users\\arisa\\Desktop\\Imp3論文用\\フィギュアfinal\\Supple.Table.S2.xlsx#Sup.Table1!B4) | [3.3](C:\\Users\\arisa\\Desktop\\Imp3論文用\\フィギュアfinal\\Supple.Table.S2.xlsx#Sup.Table1!B4) |
| *[C1r](C:\\Users\\arisa\\Desktop\\Imp3論文用\\フィギュアfinal\\Supple.Table.S2.xlsx#Sup.Table1!B4)* | [421.0](C:\\Users\\arisa\\Desktop\\Imp3論文用\\フィギュアfinal\\Supple.Table.S2.xlsx#Sup.Table1!B4) | [43.9](C:\\Users\\arisa\\Desktop\\Imp3論文用\\フィギュアfinal\\Supple.Table.S2.xlsx#Sup.Table1!B4) | [3.3](C:\\Users\\arisa\\Desktop\\Imp3論文用\\フィギュアfinal\\Supple.Table.S2.xlsx#Sup.Table1!B4) |
| *[Nts](C:\\Users\\arisa\\Desktop\\Imp3論文用\\フィギュアfinal\\Supple.Table.S2.xlsx#Sup.Table1!B4)* | [555.7](C:\\Users\\arisa\\Desktop\\Imp3論文用\\フィギュアfinal\\Supple.Table.S2.xlsx#Sup.Table1!B4) | [60.8](C:\\Users\\arisa\\Desktop\\Imp3論文用\\フィギュアfinal\\Supple.Table.S2.xlsx#Sup.Table1!B4) | [3.2](C:\\Users\\arisa\\Desktop\\Imp3論文用\\フィギュアfinal\\Supple.Table.S2.xlsx#Sup.Table1!B4) |
| *[C1qdc2](C:\\Users\\arisa\\Desktop\\Imp3論文用\\フィギュアfinal\\Supple.Table.S2.xlsx#Sup.Table1!B4)* | [112.7](C:\\Users\\arisa\\Desktop\\Imp3論文用\\フィギュアfinal\\Supple.Table.S2.xlsx#Sup.Table1!B4) | [12.9](C:\\Users\\arisa\\Desktop\\Imp3論文用\\フィギュアfinal\\Supple.Table.S2.xlsx#Sup.Table1!B4) | [3.1](C:\\Users\\arisa\\Desktop\\Imp3論文用\\フィギュアfinal\\Supple.Table.S2.xlsx#Sup.Table1!B4) |
| *[Armcx3](C:\\Users\\arisa\\Desktop\\Imp3論文用\\フィギュアfinal\\Supple.Table.S2.xlsx#Sup.Table1!B4)* | [56.7](C:\\Users\\arisa\\Desktop\\Imp3論文用\\フィギュアfinal\\Supple.Table.S2.xlsx#Sup.Table1!B4) | [6.5](C:\\Users\\arisa\\Desktop\\Imp3論文用\\フィギュアfinal\\Supple.Table.S2.xlsx#Sup.Table1!B4) | [3.1](C:\\Users\\arisa\\Desktop\\Imp3論文用\\フィギュアfinal\\Supple.Table.S2.xlsx#Sup.Table1!B4) |
| *[BC014699](C:\\Users\\arisa\\Desktop\\Imp3論文用\\フィギュアfinal\\Supple.Table.S2.xlsx#Sup.Table1!B4)* | [34.5](C:\\Users\\arisa\\Desktop\\Imp3論文用\\フィギュアfinal\\Supple.Table.S2.xlsx#Sup.Table1!B4) | [4.1](C:\\Users\\arisa\\Desktop\\Imp3論文用\\フィギュアfinal\\Supple.Table.S2.xlsx#Sup.Table1!B4) | [3.1](C:\\Users\\arisa\\Desktop\\Imp3論文用\\フィギュアfinal\\Supple.Table.S2.xlsx#Sup.Table1!B4) |
| *[Sfrp4](C:\\Users\\arisa\\Desktop\\Imp3論文用\\フィギュアfinal\\Supple.Table.S2.xlsx#Sup.Table1!B4)* | [43.1](C:\\Users\\arisa\\Desktop\\Imp3論文用\\フィギュアfinal\\Supple.Table.S2.xlsx#Sup.Table1!B4) | [5.1](C:\\Users\\arisa\\Desktop\\Imp3論文用\\フィギュアfinal\\Supple.Table.S2.xlsx#Sup.Table1!B4) | [3.1](C:\\Users\\arisa\\Desktop\\Imp3論文用\\フィギュアfinal\\Supple.Table.S2.xlsx#Sup.Table1!B4) |
| *[Ramp2](C:\\Users\\arisa\\Desktop\\Imp3論文用\\フィギュアfinal\\Supple.Table.S2.xlsx#Sup.Table1!B4)* | [54.0](C:\\Users\\arisa\\Desktop\\Imp3論文用\\フィギュアfinal\\Supple.Table.S2.xlsx#Sup.Table1!B4) | [6.4](C:\\Users\\arisa\\Desktop\\Imp3論文用\\フィギュアfinal\\Supple.Table.S2.xlsx#Sup.Table1!B4) | [3.1](C:\\Users\\arisa\\Desktop\\Imp3論文用\\フィギュアfinal\\Supple.Table.S2.xlsx#Sup.Table1!B4) |
| *[Pcsk9](C:\\Users\\arisa\\Desktop\\Imp3論文用\\フィギュアfinal\\Supple.Table.S2.xlsx#Sup.Table1!B4)* | [35.3](C:\\Users\\arisa\\Desktop\\Imp3論文用\\フィギュアfinal\\Supple.Table.S2.xlsx#Sup.Table1!B4) | [4.3](C:\\Users\\arisa\\Desktop\\Imp3論文用\\フィギュアfinal\\Supple.Table.S2.xlsx#Sup.Table1!B4) | [3.0](C:\\Users\\arisa\\Desktop\\Imp3論文用\\フィギュアfinal\\Supple.Table.S2.xlsx#Sup.Table1!B4) |
| *[Cbr1](C:\\Users\\arisa\\Desktop\\Imp3論文用\\フィギュアfinal\\Supple.Table.S2.xlsx#Sup.Table1!B4)* | [49.1](C:\\Users\\arisa\\Desktop\\Imp3論文用\\フィギュアfinal\\Supple.Table.S2.xlsx#Sup.Table1!B4) | [6.0](C:\\Users\\arisa\\Desktop\\Imp3論文用\\フィギュアfinal\\Supple.Table.S2.xlsx#Sup.Table1!B4) | [3.0](C:\\Users\\arisa\\Desktop\\Imp3論文用\\フィギュアfinal\\Supple.Table.S2.xlsx#Sup.Table1!B4) |

Those genes with an AXT/AX log2 ratio for normalized expression levels of ≥3.0 are listed.
