# Supplementary material for: Up-Regulation of Imp3 Confers In Vivo Tumorigenicity on Murine Osteosarcoma Cells
Source: PLoS One. 2012 Nov 30;7(11):e50621. doi: 10.1371/journal.pone.0050621 (PMC3511546; doi:10.1371/journal.pone.0050621)
Supplement: Table S3 — Knockdown of Imp3 in AXT cells suppresses tumorigenic activity in vivo. (DOCX) [file pone.0050621.s006.docx]

**Table S3. Knockdown of Imp3 in AXT cells suppresses tumorigenic activity in vivo.**

| [Cells ingected](C:\\Users\\arisa\\Desktop\\Imp3論文用\\フィギュアfinal\\Supple.Table.S3.xlsx#Sup.Table2!B5) | [Liver metastasis](C:\\Users\\arisa\\Desktop\\Imp3論文用\\フィギュアfinal\\Supple.Table.S3.xlsx#Sup.Table2!B5) | [Lung metastasis](C:\\Users\\arisa\\Desktop\\Imp3論文用\\フィギュアfinal\\Supple.Table.S3.xlsx#Sup.Table2!B5) |
| --- | --- | --- |
| [AXT-sh LUC](C:\\Users\\arisa\\Desktop\\Imp3論文用\\フィギュアfinal\\Supple.Table.S3.xlsx#Sup.Table2!B5) | [5/5 (100%)](C:\\Users\\arisa\\Desktop\\Imp3論文用\\フィギュアfinal\\Supple.Table.S3.xlsx#Sup.Table2!B5) | [5/5 (100%)](C:\\Users\\arisa\\Desktop\\Imp3論文用\\フィギュアfinal\\Supple.Table.S3.xlsx#Sup.Table2!B5) |
| [AXT-sh1](C:\\Users\\arisa\\Desktop\\Imp3論文用\\フィギュアfinal\\Supple.Table.S3.xlsx#Sup.Table2!B5) | [0/5 (0%)](C:\\Users\\arisa\\Desktop\\Imp3論文用\\フィギュアfinal\\Supple.Table.S3.xlsx#Sup.Table2!B5) | [0/5 (0%)](C:\\Users\\arisa\\Desktop\\Imp3論文用\\フィギュアfinal\\Supple.Table.S3.xlsx#Sup.Table2!B5) |
| [AXT-sh2](C:\\Users\\arisa\\Desktop\\Imp3論文用\\フィギュアfinal\\Supple.Table.S3.xlsx#Sup.Table2!B5) | [0/5 (0%)](C:\\Users\\arisa\\Desktop\\Imp3論文用\\フィギュアfinal\\Supple.Table.S3.xlsx#Sup.Table2!B5) | [0/5 (0%)](C:\\Users\\arisa\\Desktop\\Imp3論文用\\フィギュアfinal\\Supple.Table.S3.xlsx#Sup.Table2!B5) |

The incidence of liver and lung metastasis in mice were determined at 28 days after subcutaneous injection of AXT-shLUC, AXT-sh1, or AXT-sh2 cells.
